# Supplementary material for: Influence of Hyperproteinemia on Insect Innate Immune Function of the Circulatory System in Bombyx mori
Source: Biology (Basel). 2021 Feb 3;10(2):112. doi: 10.3390/biology10020112 (PMC7913649; doi:10.3390/biology10020112)
Supplement: Supplementary file 1 [file biology-10-00112-s001.pdf]

Supplemental Materials

Influence of Hyperproteinemia on Insect Innate Immune Function of the Circulatory System in *Bombyx mori*

Yong-Feng Wang[1,2]*, Guang Wang[1,2]*, Jiang-Lan Li[1,2], Ya-Xin Qu[1], Xin-Yin Liang[1,2], Xue-Dong Chen[1, 2], Yang-Hu Sima[1,2] and Shi-Qing Xu[1,2]

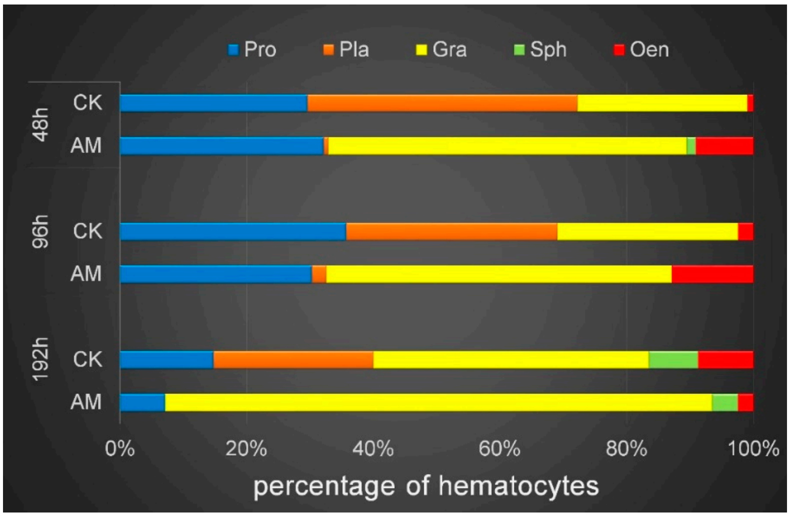

Figure S1. The proportion of hemocytes in hemolymph of silkworm after modeling. Pro, prohemocyte; Pla, plasmacyte; Gra, granulocyte; Sph, spherulocyte; Oen, oenocytoid.

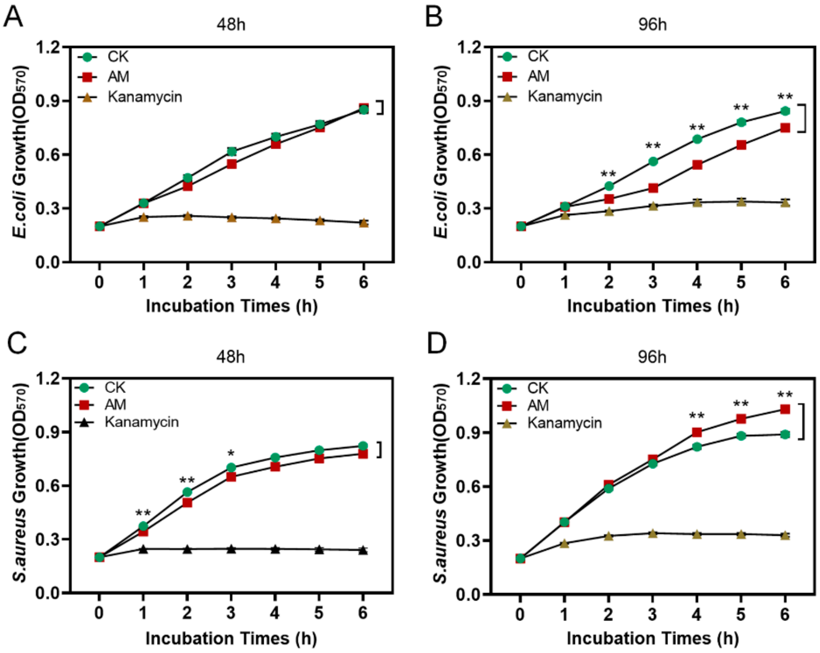

Figure S2. Antibacterial activity of high PPC silkworm cell-free hemolymph. The hemolymph of the silkworm was collected for heat treatment at 48 h and 96 h after modeling, and the supernatant was cocultured with ***E. coli*** and ***S. aureus***, and the growth of *E. coli* (**A, B**) and *S. aureus* (**C, D**) was investigated. * $p < 0.05$; ** $p < 0.01$; n.s., no significant difference between the two groups. Mean ± SEM. $n = 3$.

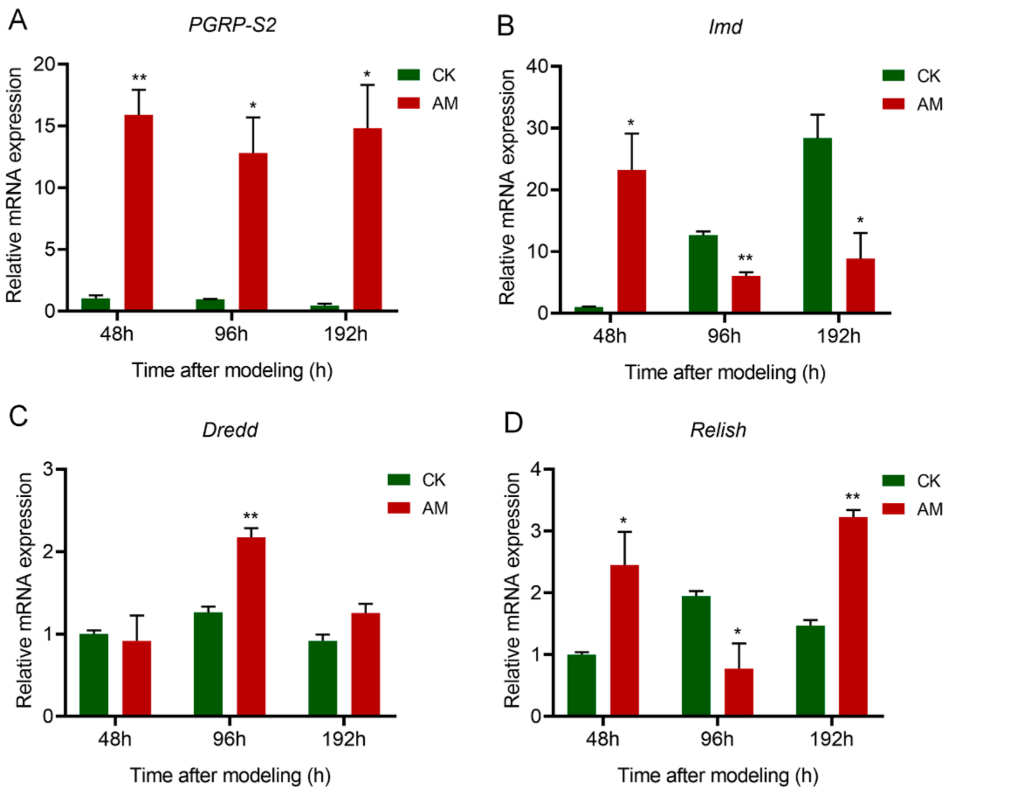

Figure S3. High PPC induces Imd signaling pathway. The mRNA levels of (**A**) *PGRP-S2* and (**B**) *Imd* and (**C**) *Dredd* and (**D**) *Relish* genes in hemocytes detected by qRT-PCR. * $p < 0.05$; ** $p < 0.01$. Mean ± SEM. $n = 3$.

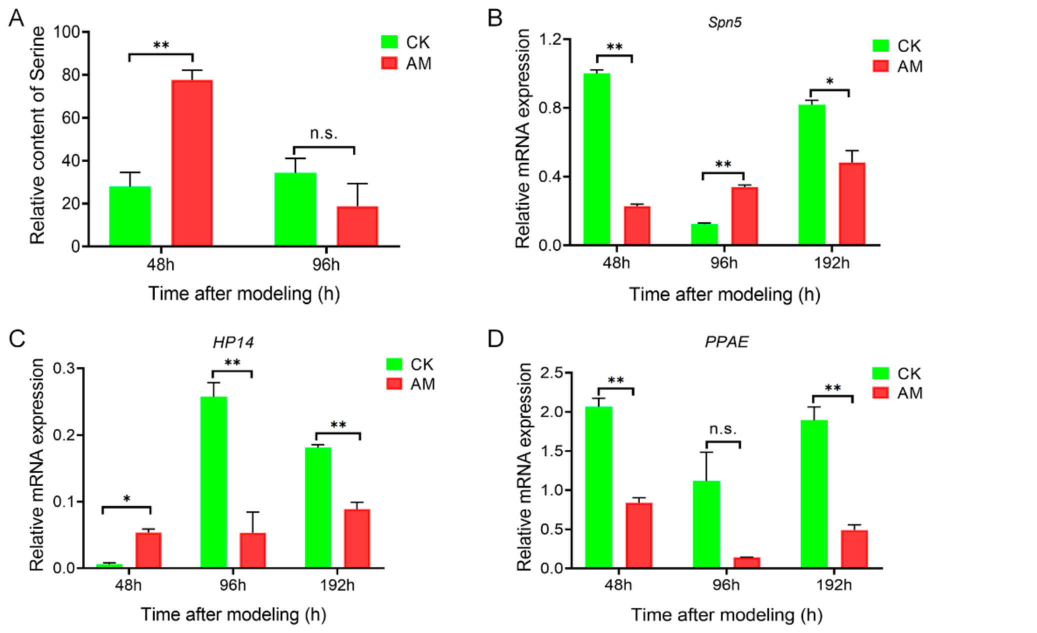

Figure S4. High PPC affects the melanization of hemolymph in silkworm. (**A**) Relative content of Serine in hemolymph at 48 h and 96 h after modeling. Serine content was determined by GC-MS method. (**B-D**) the mRNA levels of *Spn5*, *HP14* and *PPAE* in hemocytes after modeling. * $p < 0.05$; ** $p < 0.01$; n.s., no significant difference between the two groups. Mean ± SEM. $n = 3$.

Table S1. qPCR primers used in this part.

Genes	Primers	Sequences
spatzle-1	SPZ1-F	AGGAAGCATCCCGAACTGAA
	SPZ1-R	TTCTCGCCCAACTTAGCAAC
toll-like receptor 4	TLR-4-F	CATAGAGGCAGGAGTGTTTA
	TLR-4-R	ACGAAAGCGTCTTCTGGTAT
MyD88	MyD88-F	ATAGACTCGGAGGAATG
	MyD88-R	GATACTAATAGCCTGTGC
Dorsal	Dorsal-F	CAAGCAGGTCGCCGTGGTGT
	Dorsal-R	CGTCGTCCGTCAGCCTCTTCA
peptidoglycan recognition protein S2	PGRP-S2-F	TTTCATTGGCGACTTTAGGG
	PGRP-S2-R	GCTCCAGGACTTAGCGTGTT
immune deficiency	IMD-R	AGCGCCTTTGAAGCCAATGT
	IMD-F	TTTCGCCTGTGGCTACTCCA
death related ced-3/Nedd2-like protein	Dredd-F	GCAAGATGAGCCGTATGACA
	Dredd-R	AATAAGAAGTGCCTCCAGGAC
Relish	Relish -F	CCGATGAATCAGGCAACAC
	Relish -R	CCGTCTTCGAAGGCGATAG
Lebocin	Lebocin-F	GAAGGTATGTTCGCAGTGTCA
	Lebocin-R	CTTGTGTTACGGTGGCTCTC
defensin-like protein 2	Defensin 2-F	GAGCCGTGTACGAGTGAACA
	Defensin 2-R	TAAGGCCCTCCTGGATCGAA
phenoloxidase subunit 1	PPO1-F	CCTCTTGGCTGGGGTTTCTAC
	PPO1-R	TCCACGCAGTCCGTCTCG
phenoloxidase subunit 2	PPO2-F	GCTTTTGACTGAGGACTACGCC
	PPO2-R	CGCTGAGGTTCTTGAGGGGTA
ribosomal protein 49	Rp49-F	TACGGAATCCATTTGGGAGCAT
	Rp49-R	CAGGCGGTTCAAGGGTCAATC
hemolymph protease-14	HP14-F	CTTCTCCCGTAGGTTTCTGC
	HP14-R	TCCTCCAATCTCCCTCGTCT
prophenoloxidase activating enzyme	PPAE-F	GGAGGAATCTTGGATGTAGG
	PPAE-R	CTGATACGGACACGCAAT
Serpin-5	Spn5-F	AGCCAGACTTAGCCAACT
	Spn5-R	CCGATTCCGTGACCTCTA

Table S2. Differential expressed genes of Toll and Imd signaling pathway at 96 h.

Gene ID	GO_term	Go_description	KO_name	Pathway_definition
BGIBMGA012217	biological_process;;molecular_function;	BP:proteolysis;;MF:serine-type endopeptidase activity;;	MODSP	Toll and Imd signaling pathway
BGIBMGA002397			SPZ	Toll and Imd signaling pathway
BGIBMGA005140	biological_process;;molecular_function;;molecular_function;;	BP:regulation of transcription from RNA polymerase II promoter;;MF:transcription factor activity, sequence-specific DNA binding;;MF:sequence-specific DNA binding;;	FOSLN	MAPK signaling pathway - fly;;Apoptosis - fly;;Toll and Imd signaling pathway
BGIBMGA005834			NFKBIA	Leishmaniasis;;HTLV-I infection;;Chagas diseas;;IL-17 signaling pathway;;Toxoplasmosis;;Hepatitis C;;Hepatitis B;;Th17 cell differentiation;;Th1 and Th2 cell differentiation;;Influenza A;;Herpes simplex infection;;Epstein-Barr virus infection;;B cell receptor signaling pathway;;Apoptosis;;Cytosolic DNA-sensing pathway;;RIG-I-like receptor signaling pathway;;Legionellosis;;Toll-like receptor signaling pathway;;NOD-like receptor signaling pathway;;Shigellosis;;Toll and Imd signaling pathway;;Osteoclast differentiation;;Neurotrophin signaling pathway;;Small cell lung cancer;;Viral carcinogenesis;;T cell receptor signaling pathway;;Pathways in cancer;;TNF signaling pathway;;cAMP signaling pathway;;Adipocytokine signaling pathway;;Chemokine signaling pathway;;NF-kappa B signaling pathway;;Prostate cancer;;Chronic myeloid leukemia;;Epithelial cell signaling in Helicobacter pylori infection;;Measles;;Insulin resistance
BGIBMGA014370			TL	Toll and Imd signaling pathway
BGIBMGA005738			ANK	Proteoglycans in cancer;;Toll and Imd signaling pathway
BGIBMGA011607	biological_process;;biological_process;;cellular_component;;molecular_function;;molecular_function;;	BP:regulation of innate immune response;;BP:carbohydrate metabolic process;;CC:extracellular region;;MF:carbohydrate binding;;MF:hydrolase activity, hydrolyzing O-glycosyl compounds;;	GNBP3	Toll and Imd signaling pathway

BGIBMGA012215	biological_process;;molecular_function;	BP:proteolysis;;MF:serine-type endopeptidase activity;;	MODSP	Toll and Imd signaling pathway
BGIBMGA011609	biological_process;;biological_process;;biological_process;;cellular_component;;molecular_function;;molecular_function;;	BP:carbohydrate metabolic process;;BP:regulation of innate immune response;;BP:defense response to bacterium;;CC:extracellular region;;MF:carbohydrate binding;;MF:hydrolase activity, hydrolyzing O-glycosyl compounds;;	GNBP1	Toll and Imd signaling pathway
BGIBMGA005478	biological_process;;biological_process;;biological_process;;cellular_component;;molecular_function;;molecular_function;;molecular_function;;	BP:response to oxidative stress;;BP:cellular oxidant detoxification;;BP:oxidation-reduction process;;CC:integral component of membrane;;MF:heme binding;;MF:calcium ion binding;;MF:peroxidase activity;;	DUOX, THOX	MAPK signaling pathway - fly;;Toll and Imd signaling pathway
BGIBMGA006244			TL	Toll and Imd signaling pathway
BGIBMGA006017			PIRK	Toll and Imd signaling pathway
BGIBMGA006018			PIRK	Toll and Imd signaling pathway
BGIBMGA002463	biological_process;;cellular_component;;cellular_component;;molecular_function;;molecular_function;;	BP:regulation of transcription, DNA-templated;;CC:cytoplasm;;CC:nucleus;;MF:transcription factor activity, sequence-specific DNA binding;;MF:DNA binding;;	NFKB1	Leishmaniasis;;HTLV-I infection;;Chagas diseas;;IL-17 signaling pathway;;Toxoplasmosis;;Amoebiasis;;Hepatitis B;;Ras signaling pathway;;Th17 cell differentiation;;Th1 and Th2 cell differentiation;;MAPK signaling pathway;;Herpes simplex infection;;Epstein-Barr virus infection;;B cell receptor signaling pathway;;Longevity regulating pathway;;Apoptosis;;Sphingolipid signaling pathway;;Hepatitis C;;Adipocytokine signaling pathway;;Pertussis;;RIG-I-like receptor signaling pathway;;Legionellosis;;Toll-like receptor signaling pathway;;NOD-like receptor signaling pathway;;Shigellosis;;Toll and Imd signaling pathway;;Osteoclast differentiation;;Influenza A;;Cocaine addiction;;Neurotrophin signaling pathway;;Small cell lung cancer;;Tuberculosis;;MicroRNAs in cancer;;Viral carcinogenesis;;Transcriptional misregulation in can-

	**cer;;T cell receptor signaling pathway;;Pathways in cancer;;Inflammatory bowel diseas;;Salmonella infection;;TNF signaling pathway;;cAMP signaling pathway;;Fluid shear stress and atherosclerosis;;Chemokine signaling pathway;;NF-kappa B signaling pathway;;HIF-1 signaling pathway;;Cytosolic DNA-sensing pathway;;Prostate cancer;;Acute myeloid leukemia;;Pancreatic cancer;;Chronic myeloid leukemia;;Epithelial cell signaling in Helicobacter pylori infection;;PI3K-Akt signaling pathway;;Measles;;Antifolate resistance;;Prolactin signaling pathway;;Non-alcoholic fatty liver diseas;;AGE-RAGE signaling pathway in diabetic complications;;Insulin resistance**
